# Conditional effects of gaze on automatic imitation: the role of autistic traits

**DOI:** 10.1038/s41598-020-72513-6

**Published:** 2020-09-23

**Authors:** Irene Trilla, Hannah Wnendt, Isabel Dziobek

**Affiliations:** 1 Berlin School of Mind and Brain, Humboldt-Universität zu Berlin, Berlin, Germany; 2 Department of Psychology, Institute of Life Sciences, Humboldt-Universität zu Berlin, Berlin, Germany; 3 Faculty of Psychology and Neuroscience, Maastricht University, Maastricht, The Netherlands

**Keywords:** Psychology, Human behaviour

## Abstract

Establishing direct gaze has been shown to enhance the tendency to automatically imitate the other person’s actions, an effect that seems to be reduced in autism. Most previous studies, however, used experimental tasks that may have confounded the measurement of automatic imitation with spatial compatibility effects. This calls into question whether gaze cues regulate automatic imitation, or instead affect domain-general processes of response inhibition. Using a task that disentangled imitative from spatial compatibility effects, the current study re-examined the role of autistic traits on the modulation of automatic imitation by direct and averted gaze cues. While our results do not provide evidence for an overall significant influence of gaze on neither automatic imitation nor spatial compatibility, autistic traits were predictive of a reduced inhibition of imitative behaviour following averted gaze. Nonetheless, exploratory analyses suggested that the observed modulation by autistic traits may actually be better explained by the effects of concomitant social anxiety symptoms. In addition, the ethnicity of the imitated agent was identified as another potential modulator of the gaze effects on automatic imitation. Overall, our findings highlight the contextual nature of automatic imitation, but call for a reconsideration of the role of gaze on imitative behaviour.

## Introduction

Imitating the body postures, gestures and facial expressions of others is known to facilitate the understanding of their mental states^1^ and to regulate social behaviour^2–4^. While imitation may be intentional under certain circumstances, people tend to unconsciously copy observed body movements, even when they are irrelevant and could interfere with the task at hand, a process referred to as automatic imitation^5^. Automatic imitation has been shown to be an adaptive and flexible behaviour that is highly dependent on the social context. According to the social top-down response modulation (STORM) theory, automatic imitation is subtly controlled by social goals in order to promote one’s social advantage^4^.

One of the initial studies on the social modulation of automatic imitation identified gaze as an important signal that regulates imitative behaviour^6^. Together with facial expressions and body gestures, gaze cues are a rich source of non-verbal information to decode the intentions and internal states of others during social interactions^7^. Establishing direct gaze may signal social interest and intention to engage with the perceiver^8^, but it can also generate the feeling of being observed, an effect thought to promote prosocial behaviour^9^. Moreover, gaze is important for social referencing, as it shifts the attentional focus toward the gaze direction and elicits joint attention^10^.

In the context of automatic imitation, Wang et al.^6^ showed that people tend to copy observed irrelevant movements more if the imitated agent establishes direct gaze, as compared to when the agent averts the gaze away from the participant. This finding has since been replicated in successive experiments^11^, which further demonstrated that the enhancement of automatic imitation following direct gaze is related to audience effects and the signalling of affiliation intent, rather than due to gaze-triggered shifts in spatial attention. Behavioural findings are supported by neuroscientific studies showing that direct gaze enhances neural mirroring of others’ motor actions as compared to averted gaze^12,13^, and which identified the medial prefrontal cortex as a key brain region mediating the control of automatic imitation by gaze^14^.

While at first glance the evidence of gaze-triggered control of imitation seems strong, all aforementioned studies used an experimental paradigm that may have confounded the measurement of automatic imitation with more general processes of response inhibition, namely spatial compatibility. Typically, automatic imitation is assessed with stimulus–response compatibility tasks in which participants are required to perform a hand or finger movement (e.g., ‘lift index finger’) while at the same time observing a compatible (e.g., ‘index finger lift’) or incompatible (e.g., ‘middle finger lift’) action by another agent^5^. A tendency to automatically imitate the observed irrelevant movement is indicated if the participant’s performance is facilitated by observing a compatible action and/or interfered when observing an incompatible action. In many versions of this paradigm, however, the hand stimulus is displayed as a mirror view of the participant's hand, such that the observed movement is spatially aligned with the action required by the participant. In such conditions, the effects attributed to automatic imitation could also be explained in terms of the spatial compatibility between both movements^15^: the participant’s response might be facilitated not (only) because they both perform topographically similar movements (i.e. imitative compatibility), but because the stimulus and response actions involve a similar change in relative position (i.e. spatial compatibility). While many automatic imitation studies may have confounded both effects, there is evidence that imitative and spatial compatibility are two dissociable and independent processes^15,16^.

In an attempt to avoid spatial effects, the direction of the observed movement in the original automatic imitation paradigm with gaze cues was orthogonal to the response movement^6^. However, results from a recent meta-analysis indicate that orthogonal set-ups are not free from the influence of spatial compatibility effects^17^. This finding thus casts doubts on whether the gaze effects measured with this paradigm are actually affecting processes of automatic imitation or, instead, are influencing spatial compatibility.

With this confounder in mind, Marsh et al.^18^ examined the influence of gaze and group membership on automatic imitation using a paradigm that disentangled the effects of imitative and spatial compatibility. As in typical automatic imitation tasks, participants were asked to perform finger movements when observing imitatively compatible or incompatible actions. However, in half of the trials, participants observed finger movements by the left hand of the agent (i.e. mirror view), and the other half presented the agent’s right hand. While imitative and spatial compatibility coincide in the left-hand trials, inclusion of right-hand trials allows to dissociate automatic imitation from spatial compatibility. Using this set-up, Marsh et al.^18^ failed to replicate the effect of gaze on automatic imitation. Instead, social cues selectively influenced spatial compatibility, such that stronger spatial effects were found for in-group members with direct gaze and for out-group members with averted gaze. Their results thus challenge conclusions from previous research on the social modulation of automatic imitation that did not control for the independent contributions of imitative and spatial processes.

In the face of these conflicting findings, the first aim of this preregistered study was to re-examine the influence of gaze cues on automatic imitation. As in the study by Marsh et al.^18^, we used a paradigm that measured the effect of direct and averted gaze cues on automatic imitation and spatial compatibility independently. Based on the previous literature, we expected that imitative compatibility effects would be stronger following direct gaze than averted gaze. Alternatively, gaze could selectively affect spatial compatibility, such as found in Marsh et al.^18^. In this case, we would expect that direct gaze increases spatial compatibility effects as compared to averted gaze.

Second, we tested whether autistic traits modulate how gaze cues influence automatic imitation. A previous study found that individuals with autism spectrum conditions (ASC) tend to automatically imitate others’ actions, but the strength of their imitative responses is not regulated according to the gaze direction of the observed agent^19^. Importantly, this reduced contextual modulation of imitation could not be completely attributed to insensitivity to gaze cues, as direct gaze elicited alerting responses in both ASC and control samples. This and other evidence of atypical imitative behaviour in response to social signals^19,20^ may seem in conflict with studies showing intact automatic imitation in individuals with autistic traits^17,21^. In an attempt to integrate these findings, motivational accounts such as the STORM theory have proposed that, although the basic mechanisms of imitation seem to be preserved, individuals with ASC may be impaired in adjusting their imitative behaviour to the social context^4^. Accordingly, our second hypothesis predicted that the impact of gaze cues on automatic imitation would be weaker with increasing levels of autistic traits in a non-clinical sample.

In addition to the prespecified research questions, we explored if the influence of gaze on automatic imitation is conditional to other contextual factors. This question was motivated by earlier research showing that gaze cues are processed differently depending on the ethnicity and group membership of the observed face^18,22,23^. For example, in the study by Marsh et al.^18^, in-group members with direct gaze elicited stronger compatibility effects than out-group members with direct gaze. Given these context-dependent reactions to gaze, we examined whether the ethnicity of the observed agent would also shape the impact of gaze on automatic imitation.

Lastly, we explored the role of social anxiety as a predictor of the impact of gaze on automatic imitation. Social anxiety is highly prevalent in individuals with ASC, and is also characterized by impairments in social attention, such as fear and avoidance of direct eye contact^24,25^. Despite the similarities in some of the social impairments, atypical gaze patterns in social anxiety seem to be more consistent with anxiety-driven avoidance, while alterations in gaze responding in ASC have been related to reduced social motivation^25,26^. Examining whether and how social anxiety symptoms modulate the influence of gaze on automatic imitation could shed light on the mechanisms behind the observed effects in ASC.

To sum up, the current study aimed to (1) re-test the influence of direct and averted gaze on automatic imitation using a task that disentangled imitative and spatial compatibility, and (2) examine whether autistic traits modulate the influence of gaze on compatibility effects. Exploratory analyses further examined whether other social cues (i.e. ethnicity of the observed agent) and individual differences in social functioning (i.e. social anxiety) shape the impact of gaze on imitative behaviour.

## Methods

The preregistration form of this study is available at: https://osf.io/84wqe.

### Participants

Sixty participants (31 females, 28 males, 1 non-binary; M_age_ = 26.6, SD_age_ = 4.80; 58 right-handed, 2 ambidextrous) took part in this study. An a-priori power analyses using G*Power 3^27^ estimated a sample of 35 to 63 participants (alpha = 0.05, power = 0.9, within-subject repeated-measures analysis of variance) for a *ηp*^2^ reported between 0.35 (Gaze × Automatic imitation effect in Experiment 1^6^) and 0.23 (Gaze × Automatic imitation effect in the control sample^19^).

None of the participants reported current psychiatric or neurological disorders, current psychoactive medication, or history of regular substance use. Only participants with normal or corrected-to-normal vision, and who made fewer than 3 errors in the Ishihara test for colour-blindness^28^ were included.

All participants gave written informed consent and were financially remunerated for their participation. The study was conducted in compliance with the Code of Ethics of the World Medical Association (Declaration of Helsinki), and was approved by the Ethics Committee of the Psychology department at Humboldt-Universität zu Berlin.

### Materials

#### Stimuli

Clips of 5 male and 5 female actors performing direct and averted head movements were selected from the Amsterdam Dynamic Facial Expression Set (ADFES)^29^. In the direct gaze clips, the actors start with the head oriented to their left side and turn towards the observer. In the averted gaze clips, the actors start facing the observer and turn the head towards their right. The original videos were cut to 2,500 ms, and copies with vertically-flipped frames were additionally created to obtain direct and averted gaze clips in which the head moves towards the left. The size of the clips was 768 × 576 pixels. Actors maintained a neutral expression in all clips. Two of the identities (1 male, 1 female) were presented in the practice trials, and the remaining 8 identities were used for the experimental trials. For each gender, half of the actors were of Northern-European descent (white), and half were Mediterranean (Turkish or Moroccan; dark-skinned).

**Figure 1:**
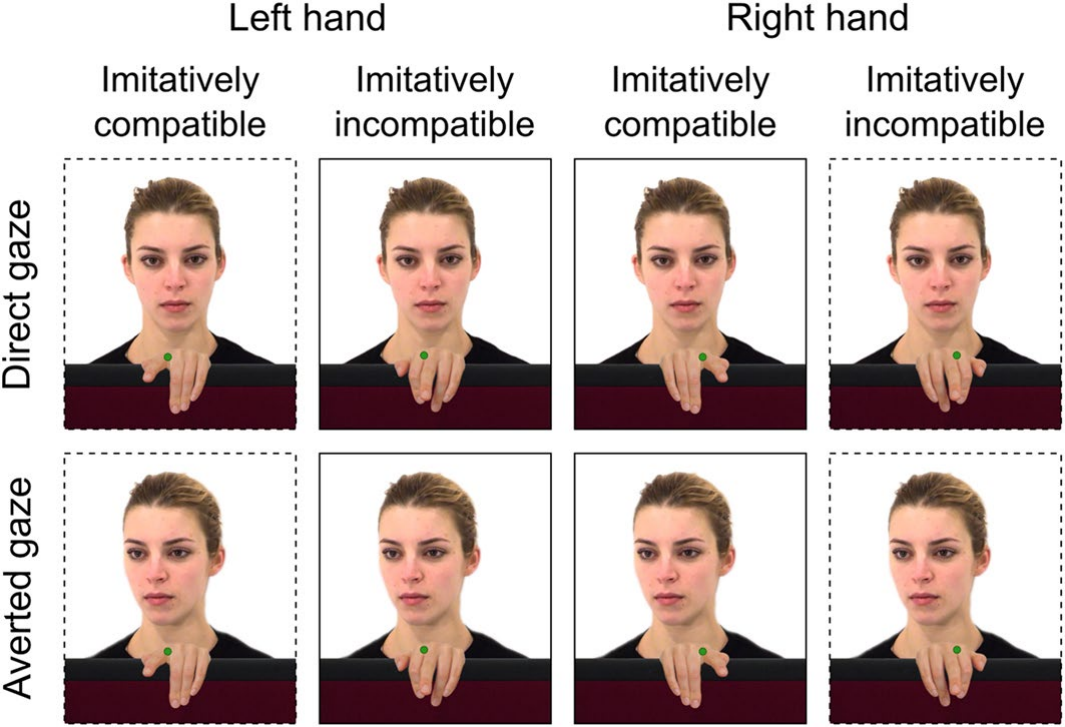
Experimental conditions of the automatic imitation task. Three factors were manipulated following a full factorial within-subject design: Gaze (direct, averted), Imitative compatibility (compatible, incompatible) and Spatial compatibility (compatible, incompatible). Dashed frames indicate spatially compatible conditions; solid frames indicate spatially incompatible conditions. In this example, participants would be required to lift their right index finger in response to a green dot, and the right middle finger in response to a purple dot.

Gaze clips were combined with hand stimuli (Fig. 1). Different female and male right hands were photographed to create the frames that would be sequentially presented in the automatic imitation task to simulate index and middle finger movements. In total, seven frames were obtained for each hand: the hand in resting position on a vertical panel, the hand with the index finger fully lifted, the hand with the middle finger fully lifted, and two intermediate positions for each of the two finger movements. Hand images were paired with the gaze stimuli based on the physical attributes of the actors and hands. All hand images were edited to match the size and skin tone to each actor, and were flipped to obtain both right- and left-hand stimuli.

#### Automatic imitation task

A stimulus–response compatibility paradigm was used to measure the effects of gaze on imitative and spatial compatibility. Participants were required to lift the index or middle finger of their right hand in response to colour cues, while at the same time observing imitatively congruent (e.g., index finger lift when prompted to lift the index finger) or incongruent actions (e.g., middle finger lift when prompted to lift the index finger). The irrelevant finger movements were performed by actors who had previously directed the gaze towards the participant (direct gaze condition) or away from them (averted gaze condition). To dissociate imitative and spatial compatibility effects, half of the trials presented the left hand of the actors, and the other half showed their right hand^15^. With this set-up, imitative and spatial compatibility overlapped in the left-hand trials (i.e. mirror view), but these effects were disentangled in the right-hand trials (Fig. 1).

**Figure 2:**
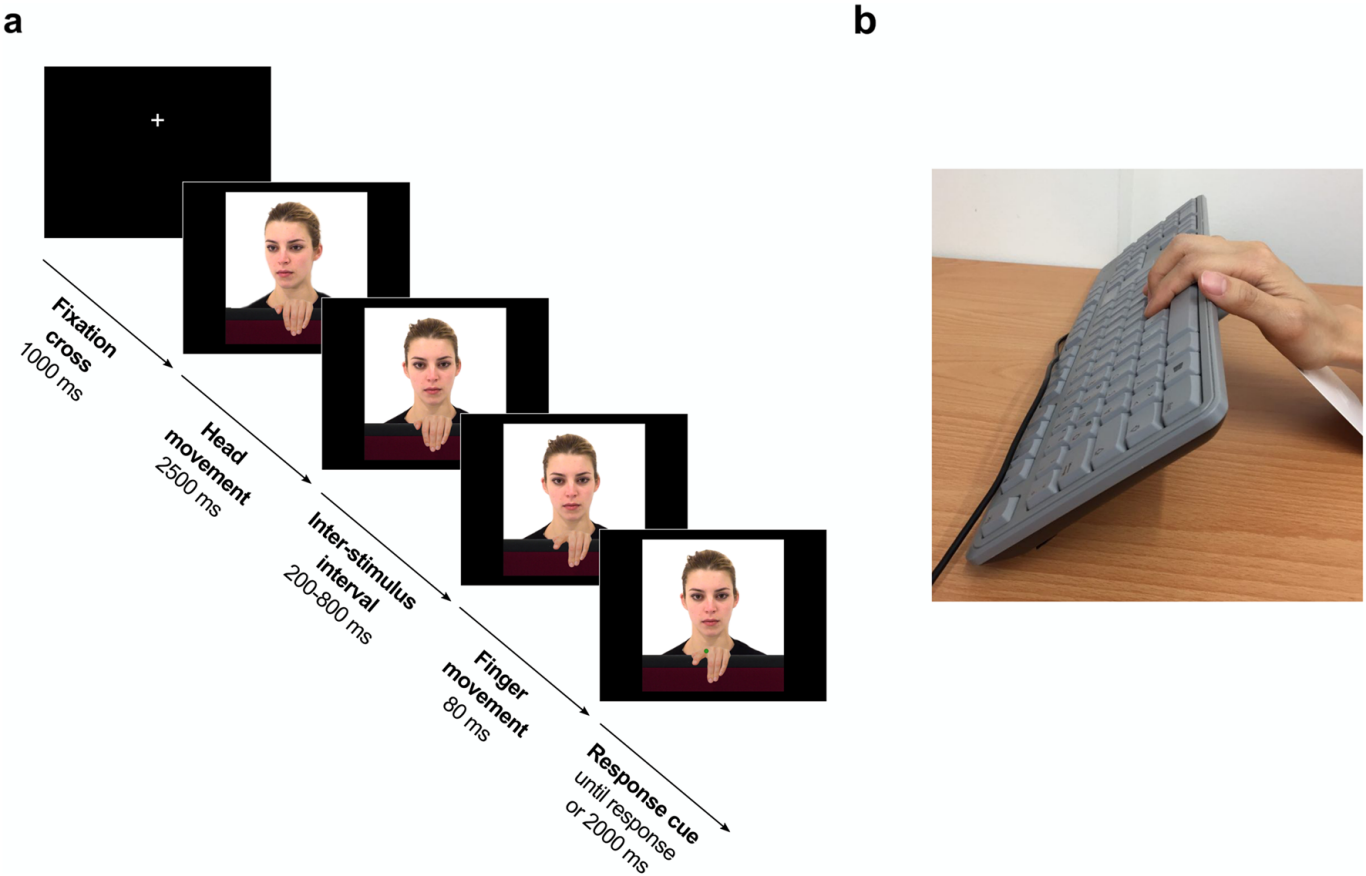
(**a**) Trial sequence of the automatic imitation task. This example represents a direct gaze, left-hand trial. (**b**) Keyboard set-up used to match the position of the participant’s hand to the orientation of the hand stimuli displayed.

Each trial of the task started with a fixation cross located at the height of the actors’ eye area for 1,000 ms (Fig. 2a). Next, participants observed a 2,500-ms clip of a direct or an averted head movement, with the actor’s hand in resting position. To avoid anticipatory responses, the final frame of the gaze clip with the resting hand remained static for a variable duration selected randomly between 200 and 800 ms. Three finger movement frames were then presented sequentially for 34 ms to induce the apparent motion of an index or middle finger lift. Eighty milliseconds after the movement onset, a purple or a green dot appeared superimposed between the actor’s index and middle finger knuckles to cue the participant’s required response. The stimulus–response correspondence (i.e. green dot = “lift index finger”, purple dot = “lift middle finger”, or vice versa) was counterbalanced across participants. An asynchronous onset of the response cue with respect to the irrelevant finger movement has been shown to facilitate imitative compatibility effects^15^. The last frame of the finger movement remained onscreen until the participant made a response, or after 2,000 ms.

#### Autistic traits and social anxiety questionnaires

The Autism Spectrum Quotient (AQ) was used to measure individual differences in autistic traits^30^. The short German version of the AQ^31^ consists of 33 items assessing different aspects related with ASC (e.g., social and communication skills, imagination and attention). The AQ has been shown to have good test–retest reliability and inter-rater reliability^30^ as well as good discriminative validity^32^.

Social anxiety was assessed with the Social Interaction Anxiety Scale (SIAS)^33^ and the Social Phobia Scale (SPS)^33^. These are widely used self-report scales that evaluate two different categories of feared situations in social anxiety disorders: those related to being observed by others (SPS), and those related to social interaction (SIAS). Finally, the German version of the Gaze Anxiety Rating Scale (GARS) was used to measure self-reported fear and avoidance of making eye contact in social situations^34^.

The internal consistency of all questionnaires in our sample was high, as indexed by Cronbach’s *α* > 0.82. Descriptive statistics for all questionnaire scores, Cronbach’s *α* and the correlations between autistic traits and social anxiety measures are available in the supplementary Table S8.

### Procedure

The testing session started with the automatic imitation task. MATLAB R2016b (The MathWorks, Inc., Natick, Massachusetts, United States) and the Psychophysics Toolbox extension^35,36^ were used to present the stimuli and collect the responses. Participants were instructed to pay attention to the head movement of the actors, and to perform the corresponding finger action at the appearance of the response cue. They were encouraged to respond as fast as possible without sacrificing accuracy. Throughout the task, participants held down two keyboard keys (“n” and “m”, marked with a green and a purple sticker) with the index and middle finger of their right hand. The key released upon making a finger lift indicated the participant’s response. Reaction times were recorded from the onset of the response cue until the key release. To ensure spatial (in)compatibility between the observed and performed movements, the position of the participant’s hand was matched to the orientation of the hand presented on the screen by placing the keyboard vertically at a 45º angle (Fig. 2b).

After the instructions, participants completed 10 practice trials with accuracy feedback to train the mapping between the colour of the response cue and the finger responses. If participants made more than two errors, additional trials were performed until a cumulative accuracy of 70% was reached. On average, participants completed 10.08 (*SD* = 0.33) practice trials.

The experimental phase consisted of 256 trials (32 trials per condition) without feedback, divided into 4 blocks. Each block comprised 64 trials, balanced for the colour of the response cue (green, purple), imitative compatibility (compatible, incompatible), the observed finger movement (index finger lift, middle finger lift) and the identity of the actor. Across all blocks, trials were also balanced for the hand presented (right hand, left hand) and the direction of the head movement of the gaze clips (towards the right, towards the left). All trials within a block presented the same hand of the actors. The order of hand blocks (‘*right-left-right-left’* or ‘*left-right-left-right’*) was counterbalanced across participants. Trials within blocks were randomly ordered. Participants could take a short break between blocks.

After the automatic imitation task, participants were asked to rate on a 5-point scale (0 = not at all, 4 = a lot) whether they generally felt “observed”, “ignored”, “connected”, “confirmed”, “rejected”, “put under pressure”, “relieved” and “distracted” when watching the clips of the actors directing the gaze towards them, and the clips of the actors directing the gaze away from them. These ratings aimed to assess the meaning that participants attributed to direct and averted gaze signals. Lastly, participants provided basic demographic information (age, gender, occupation and level of education) and completed the questionnaires assessing autistic traits and social anxiety, which were implemented in SoSci Survey^37^.

### Statistical analysis

All statistical analyses were conducted in R (R Core Team, 2016) and R studio^38^. Data and code necessary to reproduce all analyses reported here are available at https://osf.io/9gku6/.

#### Data exclusion

Reaction times (RT) and error rates were assessed independently as measures of performance. For RT analyses, trials were excluded if no response was made, if the response was incorrect, or if RTs were smaller than 200 ms or greater than 1,000 ms. To minimize the effect of outliers, trials that deviated 1.5 times from the interquartile range of RTs within each condition and participant were further excluded. We chose the interquartile range instead of the standard deviation as a measure of dispersion for outlier detection as it is more robust against extreme values and non-normality. On average, 8.55% (SD = 7.41) of the trials were excluded per participant. For error rate analyses, only trials in which no response was made within the 2,000 ms-response window were excluded. This led to a rejection of 0.05% (SD = 0.18) of trials per participant.

#### Confirmatory analyses

Generalized linear mixed-effect models (GLMM) on single-trial data were used to test our hypotheses. Our original plan was to conduct analyses of variance on aggregate data (i.e. on the mean RT and error rates for each condition and participant) to follow the statistical methods used in previous studies (see preregistration at https://osf.io/84wqe). However, GLMM provides additional advantages as they allow to: (1) account for random effects for both participants and stimuli, so more of the error can be modelled, (2) fit the data of individual trials instead of the means for each participant, which gives more statistical power, (3) accommodate missing data, so we could include data of all participants irrespective of the number excluded trials, and (4) test the effect of continuous variables, such as AQ scores^39^. Moreover, GLMMs allowed us to specify the distribution of the dependent variable to match the distributional properties of raw RT and error rate data^40^. This facilitates interpretability and comparison of results to previous studies that analysed untransformed RT. Unless otherwise specified below, results from the GLMMs reported here led to the same conclusions as the preregistered analyses, which can be found in the supplementary material.

The GLMMs on RT and error rates data included the following fixed effects: main effects of ‘Imitative compatibility’ (2 levels: compatible, incompatible), ‘Spatial compatibility’ (2 levels: compatible, incompatible), ‘Gaze’ (2 levels: direct, averted) and ‘AQ’ (continuous variable); the 2-way interactions ‘Gaze*Imitative’, ‘Gaze*Spatial’, ‘AQ*Imitative’, ‘AQ*Spatial’, and ‘AQ*Gaze’; and the 3-way interactions ‘AQ*Gaze*Imitative’ and ‘AQ*Gaze*Spatial’.

To simplify the pattern of results, we restricted the description of results to: (1) the main effects of imitative and spatial compatibility, which would indicate the occurrence of compatibility effects, and the main effect of gaze, which would suggest that gaze cues were processed; (2) the two-way interaction ‘Gaze*Imitative’ and ‘Gaze*Spatial, which would show that the degree of the corresponding compatibility effect depends on the gaze direction of the observed agent; and (3) the 3-way interactions ‘AQ*Gaze*Imitative’ and ‘AQ*Gaze*Spatial’, which would suggest that autistic traits modulate the influence of gaze on imitative (or spatial) compatibility.

Fixed effects with categorical predictors were tested using effect coding contrasts, and continuous predictors (i.e. AQ) were mean-centred. To account for non-independencies in the data, all GLMMs included by-participant and by-stimulus random intercepts. The levels of the factor ‘Stimulus’ corresponded to each of the possible combinations of the actor presented in the gaze clips, the direction of their head movement (turn towards the right side, turn towards the left side), the finger that the actor lifted (index finger, middle finger) and the colour of the response cue (green, purple).

GLMMs that included RT as dependent variable used an Inverse Gaussian distribution with Identity link function. The Inverse Gaussian is a right skewed unimodal distribution with continuous responses greater than or equal to 0 that reproduces the distributional shape of raw RT^40^. This was chosen over other possible distributions (i.e. Gaussian and Gamma) on the basis of model comparisons using AIC and BIC values and likelihood-ratio tests. The Identity link function was selected as we assumed that our manipulations linearly affected the RT, rather than some function of RT^40^. This assumption underlies experiments based on mental chronometry, and is inherent when using linear regression or linear mixed models. A mixed-effect logistic regression with a binomial distribution was conducted for error rate data to adhere to the binary nature of this variable (correct response, incorrect response).

For all models, *p*-values were calculated using Wald-statistics approximation. Statistical threshold was set at *p* < 0.05 and tests were two-tailed.

#### Exploratory analyses

Exploratory analyses were conducted on RT data, as this was a more sensitive measure than error rates. To ease comprehensibility, the statistical procedure applied for each exploratory analysis is described in the corresponding Results subsection.

## Results

### Confirmatory analyses

The mean and standard deviation of RT and error rates for each condition, as well as the complete GLMM statistics, are presented in the supplementary Tables S2 and S3.

#### Reaction times

**Figure 3:**
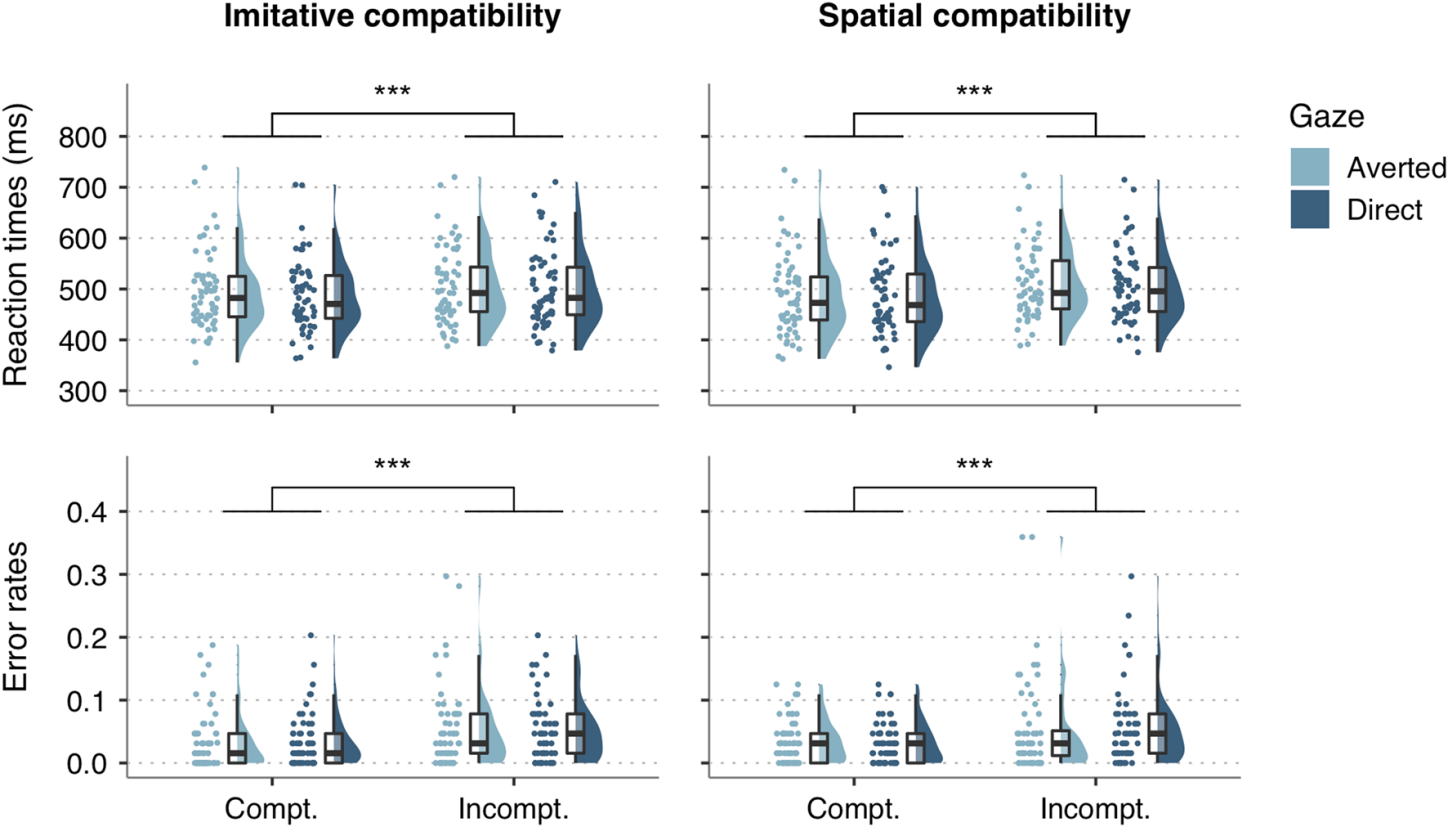
Raincloud plots of reaction times (upper panels) and error rates (lower panels) of imitatively compatible and incompatible trials (left panels), and of spatially compatible and incompatible trials (right panels), for both direct gaze (dark blue) and averted gaze (light blue) conditions. Each point represents the average reaction time or error rate of an individual participant for the corresponding condition. ***p < 0.001.

The GLMM on RT data confirmed the occurrence of both imitative compatibility, *b* = 10.23, 95% *CI* = [7.57, 12.90], *SE* = 1.36, *t* = 7.53,* p* < 0.001, and spatial compatibility effects, *b* = 22.31, *95% CI* = [19.63, 24.99], *SE* = 1.37, *t* = 16.34,* p* < 0.001 (Fig. 3). That is, participants were faster to perform correct finger movements when they observed an imitatively compatible (*M* = 489.44, *SE* = 1.40) than an imitatively incompatible action (*M* = 501.46, *SE* = 1.54). Similarly, participants responded faster to a spatially compatible action (*M* = 485.56, *SE* = 1.52) compared to a spatially incompatible action (*M* = 505.53, *SE* = 1.40). The main effect of gaze was also statistically significant, *b* = − 4.62, *95% CI* = [− 7.29, − 1.96], *SE* = 1.36, *t* = − 3.41,* p* = 0.001, indicating that participants responded faster following direct gaze (*M* = 492.95, *SE* = 1.48) than after averted gaze (*M* = 497.83, *SE* = 1.47).

**Figure 4:**
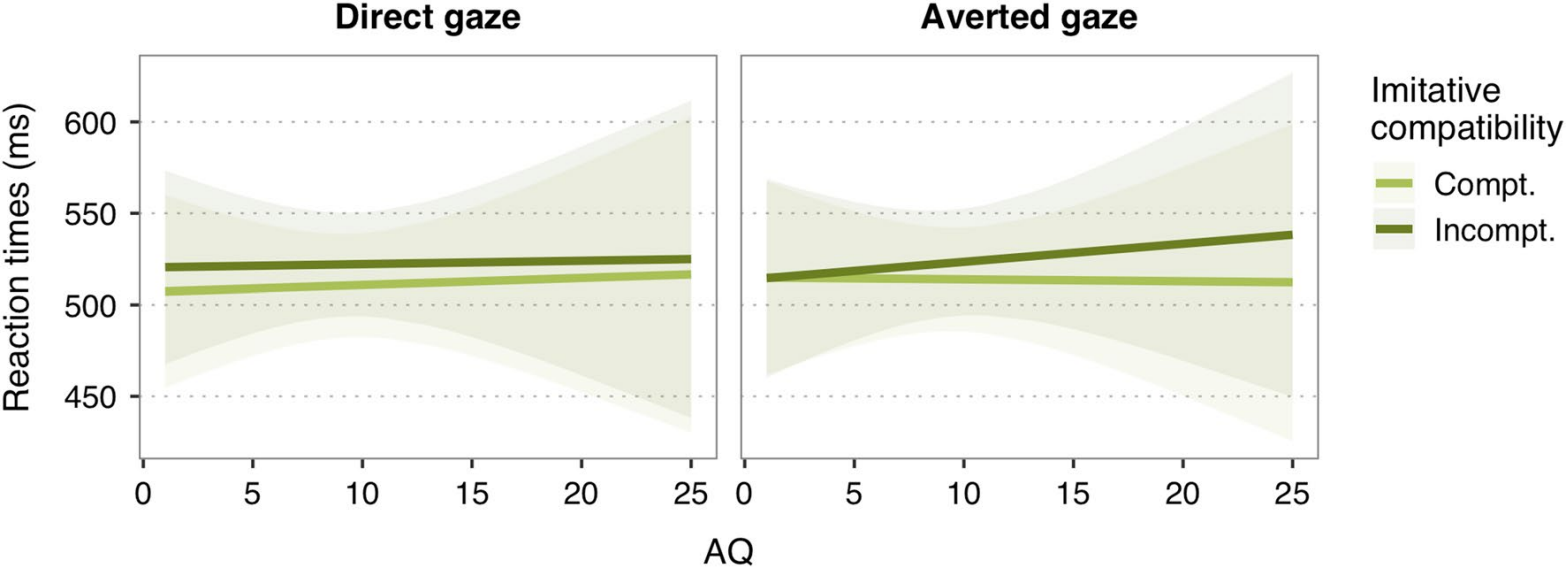
Predicted effects of autistic traits (as measured by the Autism Spectrum Quotient; AQ) on reaction times for imitatively compatible (light green line) and imitatively incompatible trials (dark green line), for direct gaze (left panel) and averted gaze (right panel) conditions. For illustration purposes, raw AQ scores are shown in the x-axis, but note that AQ scores were mean-centred before conducting the GLMM. Shaded areas indicate 95% CI.

Gaze did not significantly interact with neither imitative compatibility, *b* = 2.51, *95% CI* = [− 2.75, 7.78], *SE* = 2.69, *t* = 0.93,* p* = 0.35, nor spatial compatibility, *b* = − 5.10, *95% CI* = [− 10.44, 0.23], *SE* = 2.72, *t* = − 1.87,* p* = 0.06. The 3-way interaction between spatial compatibility, gaze and AQ was also not significant, *b* = 0.16, *95% CI* = [− 0.83, 1.14], *SE* = 0.50, *t* = 0.31,* p* = 0.75. However, AQ significantly modulated the effect of gaze on imitative compatibility, *b* = − 1.29, *95% CI* = [− 2.28, − 0.31], *SE* = 0.50, *t* = − 2.59,* p* = 0.01. To further disentangle this 3-way interaction, we tested the conditional effects of AQ on imitative compatibility for direct gaze and averted gaze conditions separately. As seen in Fig. 4, the imitative effect following averted gaze, i.e. the difference in RT between imitatively incompatible (slope = 0.85) and compatible trials (slope = − 0.23), was significantly stronger with increasing AQ scores, *b* = 1.08, *95% CI* = [0.37, 1.79], *SE* = 0.36, *t* = 2.97,* p* = 0.003. In other words, individuals with fewer autistic traits seemed to show a lower tendency to imitate the actors with averted gaze than individuals with higher autistic traits. The influence of AQ on imitative compatibility following direct gaze (slope _Incomp_ = 0.12; slope _Comp_ = 0.39) was not significant, *b* = − 0.27, *95% CI* = [− 0.97, 0.42], *SE* = 0.35, *t* = − 0.78,* p* = 0.44.

In the preregistered analysis, the AQ modulation of the gaze effects on imitative compatibility did not reach statistical significance (see supplementary Table S1). Note, however, that the originally planned approach is not directly comparable to the one reported here. For the preregistered analysis, we first computed imitative compatibility scores for each participant by subtracting the mean RT of imitatively compatible trials from the mean RT of imitatively incompatible trials. Imitative compatibility scores were then used as the dependent variable in a linear mixed model testing the interaction between AQ and gaze. The preregistered analysis thus examined whether the difference in the level of imitation following direct gaze compared to averted gaze varied across AQ scores, a 2-way interaction that was not statistically significant, *b* = − 0.97, *95% CI* = [− 2.36, 0.41], *SE* = 0.71, *t* = − 1.38,* p* = 0.17. In contrast, the model described here tested whether the influence of autistic traits on imitatively compatible and incompatible trials differed for direct gaze and averted gaze conditions.

#### Error rates

The GLMM on error rates yielded significant main effects of imitative compatibility, *OR* = 1.58, *95% CI* = [1.35, 1.85], *SE* = 0.08, *t* = 5.59,* p* < 0.001, and spatial compatibility, *OR* = 1.70, *95% CI* = [1.45, 2.00], *SE* = 0.08, *t* = 6.46,* p* < 0.001. Compatibility effects indicate that error rates were higher in imitatively incompatible (*M* = 0.05, *SE* = 0.004) and spatially incompatible trials (*M* = 0.05, *SE* = 0.004) than in imitatively compatible (*M* = 0.04, *SE* = 0.003) and spatially compatible trials (*M* = 0.03, *SE* = 0.003), respectively (Fig. 3). None of the remaining main effects or interactions were statistically significant (all *p* > 0.07).

### Exploratory analyses

#### General compatibility

To make our results comparable to previous automatic imitation studies in which imitative and spatial compatibility were confounded, we ran an additional GLMM on RT data from the subset of trials in which spatial and imitative compatibility overlap (i.e. left-hand trials)^18^. This model yielded a main effect of general compatibility, *b* = 33.11, *95% CI* = [29.45, 36.77], *SE* = 1.87, *t* = 17.73,* p* < 0.001*,* with faster correct responses in compatible (*M* = 481.00, *SE* = 1.98) than in incompatible trials (*M* = 513.15, *SE* = 117.40). The main effect of gaze was also significant, *b* = − 4.95, *95% CI* = [− 8.58, − 1.33], *SE* = 1.85, *t* = − 2.68,* p* = 0.007*,* showing that responses following direct gaze (*M* = 494.21, *SE* = 2.00) were faster than following averted gaze (*M* = 499.10, *SE* = 2.06). Neither the interaction between gaze and general compatibility, *b* = − 2.65, *95% CI* = [− 9.87, 4.58], *SE* = 3.69, *t* = − 0.72,* p* = 0.47, nor the predicted three-way interaction with AQ*, b* = − 1.34, *95% CI* = [− 2.68–0.01], *SE* = 0.69, *t* = − 1.95,* p* = 0.05, reached the significance threshold. See supplementary Table S4 for the full model statistics.

#### Interaction between gaze and the ethnicity of the imitated agent

To explore whether participants reacted differently to the gaze cues of Northern-European actors compared to Mediterranean actors, we ran a GLMM with RT data that included the same predictors as in the confirmatory analysis, except that the variable ‘AQ’ was replaced by the 2-level categorical factor ‘Ethnicity’. The model specification and full statistics are available in the supplementary Table S5.

**Figure 5:**
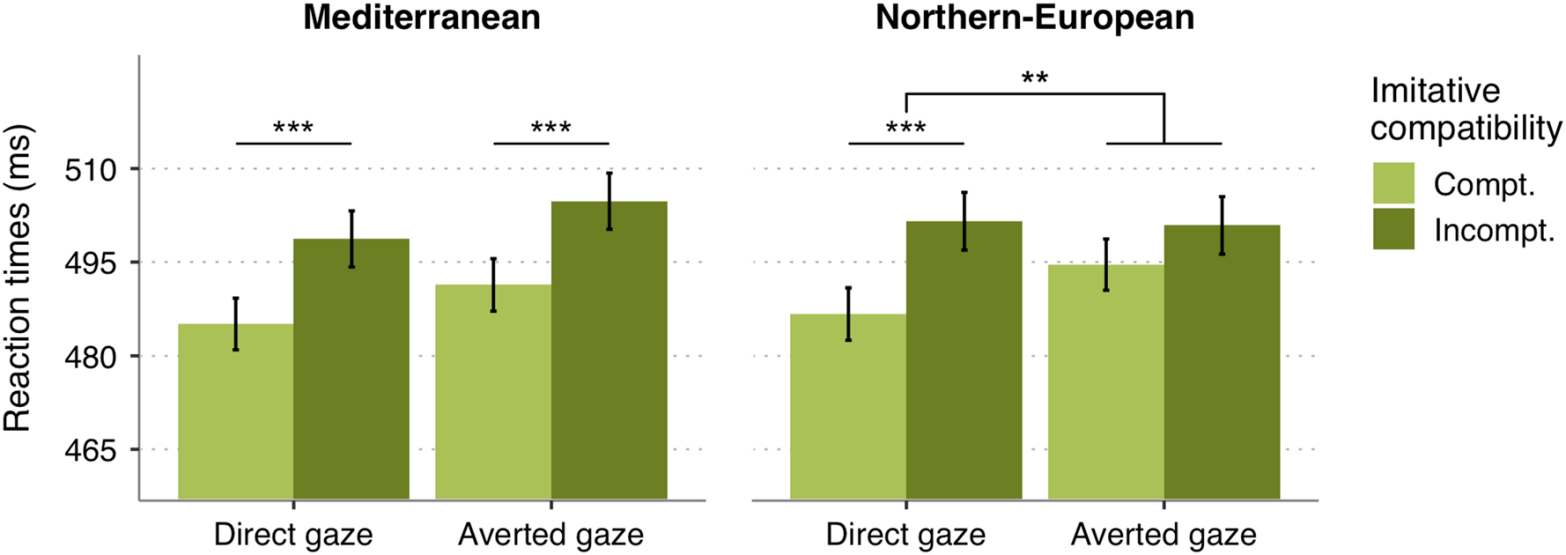
Mean reaction times of imitatively compatible (light green bars) and incompatible trials (dark green bars) as a function of gaze cues and ethnicity of the observed actors. Error bars represent within-subject 95% CI. ***p < 0.001, **p < 0.01.

As in all previous models, the main effects of imitative compatibility, spatial compatibility and gaze were significant (all *p* < 0.002). In addition, we found a significant 3-way interaction between the actors’ ethnicity, gaze and imitative compatibility, *b* = 13.72, *95% CI* = [4.20, 23.25], *SE* = 4.86, *t* = 2.82,* p* = 0.005 (Fig. 5). Follow-up analyses revealed a significant effect of gaze on imitative compatibility for Northern-European actors, *b* = 9.41, *95% CI* = [2.29, 16.54], *SE* = 3.64, *t* = 2.59,* p* = 0.01, but not for Mediterranean actors,* b* = − 4.60, *95% CI* = [− 11.64, 2.44], *SE* = 3.59, *t* = − 1.28,* p* = 0.20. Specifically, Northern-European identities elicited imitative effects only when they established direct gaze, *M*_Incomp-Compt._ = 13.45, *SE* = 2.60, *z* = 5.18, *p* < 0.001, but not with averted gaze, *M*_Incomp-Compt._ = 4.14, *SE* = 2.60, *z* = 1.60, *p* = 0.11. In contrast, Mediterranean-looking actors triggered imitative compatibility effects in both direct gaze, *M*_Incomp-Compt._ = 9.59, *SE* = 2.60, *z* = 3.70, *p* < 0.001, and averted gaze conditions, *M*_Incomp-Compt._ = 14.00, *SE* = 2.64, z = 5.23, *p* < 0.001.

#### Social anxiety

To explore whether symptoms of social anxiety modulated the influence of gaze on automatic imitation, we ran three GLMMs on RT data that included the same predictors as in the confirmatory analyses, except that the variable ‘AQ’ was replaced by the scores of each of the social anxiety questionnaires (SIAS, SPS and GARS), respectively. The supplementary Table S6 includes the specifications and results of these models.

In addition to the significant main effects of imitative compatibility, spatial compatibility and gaze (all *p* < 0.002), the model with SIAS as predictor revealed a significant 3-way interaction between SIAS, gaze and imitative compatibility,* b* = − 0.68, *95% CI* = [− 1.09, − 0.26], *SE* = 0.21, *t* = − 3.19,* p* = 0.001. Follow-up analyses showed that, as in the case of AQ, the imitative effect in the averted gaze condition was significantly stronger with increasing SIAS scores, *b* = 0.37, *95% CI* = [0.07, 0.67], *SE* = 0.15, *t* = 2.40,* p* = 0.02. Conversely, imitative effects following direct gaze were weaker as a function of SIAS scores, *b* = − 0.32, *95% CI* = [− 0.62, − 0.02], *SE* = 0.15, *t* = − 2.12,* p* = 0.03. The 3-way interaction between SIAS, gaze and spatial compatibility was not significant,* b* = 0.22, *95% CI* = [− 0.20, 0.64], *SE* = 0.21, *t* = 1.04,* p* = 0.30.

A similar pattern of results was found in the GLMMs with the other two measures of social anxiety: both the 3-way interaction between imitative compatibility, gaze and SPS, *b* = − 0.72, *95% CI* = [− 1.32, − 0.13], *SE* = 0.30, *t* = − 2.38,* p* = 0.02, and between imitative compatibility, gaze and GARS, *b* = − 0.36, *95% CI* = [− 0.70 to − 0.01], *SE* = 0.17, *t* = − 2.05,* p* = 0.04, were significant. No significant interactions were found with spatial compatibility (all *p* > 0.35).

Given that autistic traits and social anxiety seem to modulate the gaze effects on automatic imitation in a similar fashion, and that AQ scores correlated positively with social anxiety measures, all *r*_s_(60) > 0.29, *p* < 0.05 (Table S8), we conducted an additional GLMM to explore whether the observed modulation by AQ could be (partly) attributed to the influence of comorbid social anxiety symptoms. This model included both the 3-way interaction ‘AQ*Gaze*Imitative’ and ‘SIAS*Gaze*Imitative’, as well as the corresponding 2-way interactions and main effects for all factors (see supplementary Table S7). To avoid overfitting, this model tested the effects on imitative compatibility, but not on spatial compatibility. Results showed that, when accounting for the influence of SIAS, the estimated predictive value of the interaction between AQ, gaze and imitative compatibility was lower and no longer statistically significant, *b* = − 0.56, *95% CI* = [− 1.82, 0.71], *SE* = 0.65, *t* = − 0.86,* p* = 0.39. The 3-way interaction with SIAS remained significant, *b* = − 0.54, *95% CI* = [−1.08, − 0.004], *SE* = 0.27, *t* = − 1.97,* p* = 0.048.

#### Attributed meaning of gaze

Paired-samples *t*-tests were conducted to compare how participants felt in response to direct vs. averted gaze clips for each rated attribute. *P*-values were adjusted using the Holm-Bonferroni correction. Participants reported feeling significantly more observed, connected with, accepted, distracted, and pressured by the observed faces following direct gaze as compared to averted gaze (all *p* < 0.001, Cohen’s *d* > 0.50). Conversely, averted gaze clips induced more feelings of being ignored and rejected than direct gaze clips (all *p* < 0.001, Cohen’s *d* > 0.45). No significant differences were found between gaze conditions with respect to feeling relieved (*p* = 0.31, Cohen’s *d* = 0.13). Summary statistics and complete *t*-tests results are available in the supplementary Table S9.

## Discussion

The current preregistered study aimed to resolve inconsistent findings on the influence of gaze cues on automatic imitation. Using a task that disentangled imitative and spatial compatibility, we did not find significant evidence for an overall influence of direct and averted gaze on neither automatic imitation nor spatial processes. However, autistic traits predicted the degree to which participants adapted their imitative behaviour to the gaze cues, such that higher autistic traits were associated with a lower inhibition of imitative responses following averted gaze. In addition, exploratory analyses identified that the ethnicity of the imitated agent, as well as symptoms of social anxiety, may be further modulators of the gaze effects on automatic imitation.

### Why did we not replicate the effects of gaze on automatic imitation?

Several factors could explain our failure to replicate the previously reported effect of gaze on automatic imitation. First, participants may have ignored the gaze clips in our study. However, this explanation is unlikely as gaze direction had an overall influence on reaction times, such that participants responded faster following direct gaze than averted gaze. This main effect of gaze has been observed in previous studies^6,11,19^, and is thought to reflect the attention reorienting properties of gaze cues. As shown by eye-tracking data collected in a previous automatic imitation study with gaze clips, direct gaze triggers participants to look at the face region more than averted gaze^12^. In addition, participants in our study reported feeling more observed, connected and accepted when the actors established direct gaze compared to averted gaze, which induced more feelings of rejection and of being ignored. Although these subjective effects were recalled only after the task was finished, they are a further indication that gaze information was processed in accordance to the social evaluations of gaze cues^8^.

Second, it could be that gaze alone is not a powerful enough signal to regulate imitative behaviour. In our task, the actors that participants observed maintained a neutral expression throughout the gaze clips. In contrast, the agent presented in previous studies established eye contact with a small smile^6,11,12,19^. The combined product of eye contact with a smile, rather than the direct gaze itself, is what may have been interpreted as a signal of social engagement and promoted automatic imitation. In fact, according to the authors, imitative effects during pilot testing in their lab were not elicited if the model established direct gaze without a smile^11^. Similarly, even though Marsh et al.^18^ did not find effects on automatic imitation, the influence of gaze on spatial compatibility was conditional upon the group membership of the observed agent. This further suggests that direct and averted gaze may not uniformly influence compatibility effects, but rather the (social) meaning and impact of gaze critically depends on other contextual factors^7^.

To explore this idea, we examined whether participants in our study reacted differently to the gaze cues depending on the ethnicity of the observed actors. Results showed that the degree of automatic imitation was significantly influenced by the gaze cues of Northern-European actors, but not of Mediterranean actors. Specifically, Northern-European identities were only imitated following direct gaze, but not after averted gaze, while Mediterranean actors were automatically imitated regardless of their gaze direction. Although we did not register the ethnicity of our participants, study samples tested in our lab are typically composed of a majority of European decent. Under this assumption, our observations are congruent with gaze cuing studies showing a higher sensitivity to gaze cues of own-race^22,23,41^ and high-status faces^42,43^. Due to the exploratory nature of this finding, the combined role of gaze and ethnicity on automatic imitation should be taken carefully until further replication. Nevertheless, our results provide tentative evidence that, as speculated above, the influence of gaze on automatic imitation is dependent on other contextual factors.

Our experimental design also deviated from other studies in that the observed head movements and motor actions were performed by eight different identities. In contrast, most previous paradigms presented the same female actress in all trials^6,11^. Just by the mere-exposure effect, i.e. the increasing preference for a stimulus with repeated exposure^44^, participants in earlier studies may have developed increased liking and sympathy for the actress over trials. As familiarity^45^ and holding a positive attitude towards the other^43^ seems to enhance sensitivity to gaze cues, an increased interest for the observed agent could have made their gazing behaviour more relevant to the participant. This, in turn, may have increased the likelihood to observe gaze effects in previous studies.

Lastly, it could be that our stimuli lacked ecological validity. In a recent TMS study, observation of hand movements following direct gaze elicited stronger cortical motor resonance than after averted gaze in a live two-person context, but not when the gaze cues and hand actions were presented via video-taped recordings^13^. Though video stimuli as used in the current and prior studies allow for better experimental control, they lack the richness and social meaning inherent in real human interactions, which may hinder the motivation to pay attention to and engage with the stimuli. As such, future studies could further investigate the role of gaze on imitation in more naturalistic settings.

### Do autistic traits (and social anxiety) modulate the effects of gaze on automatic imitation?

Previous studies have shown that individuals with ASC tend to automatically imitate others’ actions^17,46^, but their imitative behaviour is not typically regulated according to the social context^19,20^. In line with this observation, we found that autistic traits in a non-clinical sample predicted the extent to which participants adapted their imitative responses to the gaze cues. However, the modulation by autistic traits reached statistical significance only in the GLMM analysis, not in the preregistered tests. Although this discrepancy could be due to the fact that mixed-effects models on single-trial data are more powerful than the preregistered analysis plan, results from post-hoc tests further challenge the value of autistic traits in predicting the impact of gaze on automatic imitation.

In particular, exploratory analyses indicated that social anxiety may modulate the effects of gaze on automatic imitation in a similar direction to autistic traits. That is, higher scores in both the AQ (autistic traits) and SIAS (social anxiety) were associated with a reduced inhibition of imitative behaviour following averted gaze, and weaker imitative responses following direct gaze, although the latter effect was significant only for SIAS. Given that previous literature typically framed the gaze effects as an enhancement of imitation following direct gaze^6,7,11^, the influence of AQ and SIAS on imitative reactions to averted gaze could seem surprising. However, if we consider this type of imitation an automatic process^5^, it is plausible that factors influencing the social modulation of imitation would mostly operate by influencing the ability to inhibit (automatic) imitative responses in situations in which such behaviour would be socially disadvantageous, rather than (or in addition to) enhancing imitation when this would lead to positive social outcomes.

Given the positive correlations between AQ scores and social anxiety measures, the question arose of whether the observed modulation by autistic traits could be related to the concomitant influence of social anxiety. In line with this hypothesis, the predictive value of autistic traits decreased and was no longer statistically significant when the effect of SIAS was accounted for. Because social anxiety symptoms are common in ASC^47^, future investigations should assess whether the reduced social modulation of imitation previously observed in individuals with ASC^19,20^ could be better explained by comorbid social anxiety. Nevertheless, the stronger weight of SIAS may also be an indication that aspects related to fear of social interactions and atypical social attention, which are symptoms measured by SIAS and shared with ASC^24,26,47^, may be more relevant to the gaze modulation of imitation than other ASC-related constructs assessed by the AQ, such as impairments in communication skills or imagination^31^. Future work in larger (sub)clinical samples is needed to disambiguate the patterns of relations between autistic traits and social anxiety underlying the influence of gaze on automatic imitation.

### Do social factors modulate automatic imitation independent of spatial compatibility?

Methodological limitations of earlier studies called into question whether social cues thought to regulate automatic imitation could be actually affecting more domain-general mechanisms of response inhibition, such as spatial compatibility^18^. In line with this idea, a recent meta-analysis of neuroimaging studies found consistent evidence for the involvement of domain-general brain networks (e.g., dorsolateral frontoparietal cortex) in the control of imitative responses, and only limited support for the engagement of domain-specific systems related to social cognition, such as the theory of mind network^48^.

In our study, none of the factors tested (i.e. gaze, ethnicity, autistic traits, social anxiety) showed any significant effects on spatial compatibility. Our data thus does not provide support to the previous observation that social cues selectively affect spatial compatibility^18^. Instead, results from this study are more consistent with the idea that gaze cues, at least in interaction with other factors, modulate automatic imitative responses, even when spatial effects are controlled for. In agreement with the domain-specific hypothesis, neurostimulation studies have implicated the right temporoparietal junction in the control of imitative responses independently of spatial compatibility effects^49^, and the medial prefrontal cortex has been identified as a central region in the social modulation of automatic imitation^14,50^.

Nevertheless, non-significant results do not prove the absence of an effect. It is possible that spatial compatibility is also modulated by social cues, but that we were unable to detect it. Because spatial effects are typically stronger in magnitude than imitative effects (here, spatial effects for RT data, *b* = 22.31, were twice as strong as imitative effects, *b* = 10.23)^15,18^, subtle changes by social cues may be more difficult to uncover. The only tentative indication of a social modulation of spatial compatibility was a close-to-significant interaction with gaze in the GLMM on RT data, with stronger spatial effects in response to averted gaze compared to direct gaze. However, this effect was in the opposite direction as hypothesized, and is not in congruence with the interaction between gaze and group membership reported in Marsh et al.^18^. Altogether, the inconsistent findings with respect to whether and how social cues impact spatial compatibility call for cautions conclusions regarding the contextual nature of domain-general compatibility effects.

## Limitations

Even though our sample size was determined based on the magnitude of the gaze effects reported in previous automatic imitation studies^6,19^, and it included at least twice as many participants, we had limited power to investigate effects of small-to-medium size, which are more likely for interactions with personality traits and contextual factors. Underpowered studies do not only limit the chance of finding an effect, but also reduce the likelihood that a statistically significant result reflects a true effect^51^. Therefore, and given the exploratory nature of some of the analyses reported here, further studies with bigger sample sizes are needed to validate the observed social modulation of imitation, as well as the role of autistic traits and social anxiety.

Moreover, this and previous studies on the effects of gaze on imitative behaviour have used tasks that measure imitation of very simple, meaningless finger or hand actions^6,11,18,19^. Though these tasks are widely used in automatic imitation research, they may not fully capture the nature of the actions and gestures that would be spontaneously imitated in real social interactions. Future research could aim to replicate the observed contextual effects of gaze with automatic imitation paradigms that build on more socially meaningful gestures, such as in^52,53^. Prospective studies would also benefit from including a baseline condition in which no gaze cue precedes the observed irrelevant finger/hand action. A comparison between each gaze condition with the baseline may shed light on whether the gaze effects are due to a stronger tendency to imitate the other’s actions following direct gaze, and/or reduced imitative responses after averted gaze.

## Conclusion

Our study highlights the importance of (preregistered) replications in psychological research. Even though the influence of gaze on automatic imitation was supported by several studies^6,11,12,14,19^, most of the successful replication attempts were conducted using the same paradigm as in the original study. Albeit direct replications are crucial to control for sampling error, partial and conceptual replications (i.e. experiments that test the same phenomenon with different methodology) are also needed to confirm the internal validity and generalizability of the findings, especially when a research topic appears to be highly sensitive to contextual factors^54^.

Results from this study indicate that the influence of gaze on automatic imitation may not be as consistent and uniform as reported in the literature. By using different stimuli and task manipulations, both Marsh et al.^18^ and the current study have helped to identify potential modulators of the gaze effects on automatic imitation that were masked before. For example, preliminary findings from our study suggest that characteristic of the imitated agent (e.g., ethnicity), as well as individual differences in social functioning (e.g., autistic traits, social anxiety), should be considered when assessing the role of gaze cues on the regulation of imitative behaviour. Moreover, our results strengthen the idea that imitative and spatial compatibility effects are dissociable processes, although more work is needed to determine how social factors affect them. Future research should systematically test the relationship between the different contextual modulators to better characterize the key elements involved in the social regulation of automatic imitation.

## Supplementary information

Supplementary Information 1.

## Data Availability

The datasets generated and/or analysed during the current study are available in the Open Science Framework repository: https://osf.io/9gku6/.
