# Supplementary material for: Conditional effects of gaze on automatic imitation: the role of autistic traits
Source: Sci Rep. 2020 Sep 23;10:15512. doi: 10.1038/s41598-020-72513-6 (PMC7511335; doi:10.1038/s41598-020-72513-6)
Supplement: Supplementary file 1 — Supplementary Information 1. [file 41598_2020_72513_MOESM1_ESM.pdf]

Conditional effects of gaze on automatic imitation: the role of autistic traits

Irene Trilla[1,2,*], Hannah Wnendt[1,3], Isabel Dziobek[1,2]

[1] Berlin School of Mind and Brain, Humboldt-Universität zu Berlin, Berlin, Germany
[2] Department of Psychology, Institute of Life Sciences, Humboldt-Universität zu Berlin, Berlin, Germany
[3] Faculty of Psychology and Neuroscience, Maastricht University, Maastricht, The Netherlands
*Correspondence: irene.trilla@hu-berlin.de

Supplementary material

Preregistered analyses

Analysis sample ... 2
Hypothesis 1: Effects of gaze on imitative and spatial compatibility ... 2
Hypothesis 2: AQ modulation of the effects of gaze on imitative and spatial compatibility ... 2
Table S1 ... 3

Confirmatory analyses

Table S2. Descriptive statistics ... 4
Table S3. GLMM ... 5

Exploratory analyses

Table S4. GLMM: General compatibility ... 6
Table S5. GLMM: Ethnicity ... 7
Table S6. GLMM: Social anxiety ... 8
Table S7. GLMM: Autistic-like traits & Social anxiety ... 9
Table S8. Questionnaires: descriptive statistics and correlations ... 10
Table S9. Attributed meaning of gaze ... 10

Data availability: data and code necessary to reproduce all analyses reported here, as well as the preregistration, and additional supplementary files, are available at: osf.io/9gku6.

Pre-registered statistical analyses

Analysis sample

Following the preregistered exclusion criteria, data from 2 participants were removed from these analyses due to rejection of more than 40% of the trials in one or more conditions. This led to an analysis sample size of 58 participants (30 females, 27 males, 1 non-binary).

Hypothesis 1: Effects of gaze on imitative and spatial compatibility

To test the effects of gaze on imitative and spatial compatibility, we computed the mean reaction times (RT) and mean error rates (ER) for each of the 8 conditions for each participant. Each dependent variable (RT, ER) was analysed with a three-way repeated-measures ANOVA with 'Gaze' (2 levels: direct, averted), 'Spatial compatibility' (2 levels: compatible, incompatible) and 'Imitative compatibility' (2 levels: compatible, incompatible) as within-subject factors.

The ANOVA on RT yielded a significant main effect of both imitative compatibility, $F(1, 57) = 22.04$, $MSE = 727.05$, $p < .001$, $\eta_{p}^{2} = .28$, and spatial compatibility, $F(1, 57) = 49.36$, $MSE = 841.68$, $p < .001$, $\eta_{p}^{2} = .46$. Specifically, participants were faster to perform the correct finger movements when they observed an imitatively compatible action ($M = 494.96$, $SD = 34.46$) as compared to an imitatively incompatible action ($M = 506.44$, $SD = 34.92$). Similarly, RT were lower for spatially compatible trials ($M = 491.10$, $SD = 32.65$) as compared to incompatible trials ($M = 510.03$, $SD = 33.47$). The main effect of gaze was also statistically significant, $F(1, 57) = 8.55$, $MSE = 307.89$, $p = .005$, $\eta_{p}^{2} = .13$, showing that participants responded faster after direct gaze ($M = 498.18$, $SD = 34.91$) than averted gaze ($M = 502.94$, $SD = 36.11$). Neither of the predicted interactions gaze*imitative compatibility, $F(1, 57) = 2.07$, $MSE = 425.33$, $p = .16$, $\eta_{p}^{2} = .04$, nor gaze*spatial compatibility, $F(1, 57) = 1.57$, $MSE = 320.49$, $p = .21$, $\eta_{p}^{2} = .03$, were statistically significant.

The repeated-measures ANOVA on ER yielded a significant main effect of imitative compatibility, $F(1, 57) = 13.66$, $MSE = 0.002$, $p < .001$, $\eta_{p}^{2} = .19$, such that participants made fewer errors in imitatively compatible trials ($M = 0.03$, $SD = 0.05$) than in incompatible trials ($M = 0.05$, $SD = 0.05$). The main effect of spatial compatibility was also significant, $F(1, 57) = 14.28$, $MSE = 0.001$, $p < .001$, $\eta_{p}^{2} = .20$, with spatially compatible trials ($M = 0.03$, $SD = 0.05$) leading to fewer errors than spatially incompatible trials ($M = 0.04$, $SD = 0.05$). None of the remaining effects reached statistical significance (all $p > .34$).

Hypothesis 2: AQ modulation of the effects of gaze on imitative and spatial compatibility

To test hypothesis 2, we first computed imitative and spatial compatibility scores for each participant and gaze condition, for both RT and ER. Compatibility scores were calculated by subtracting the mean RT [ER] of imitatively [spatially] compatible trials from the mean RT [ER] of imitative [spatially] compatible trials. Positive values indicate the occurrence of a compatibility effect (i.e. slower RT [higher ER] in incompatible trials than compatible trials).

The computed compatibility scores were fitted to linear mixed-effect models (LMM) to test the influence of autistic traits on the effect of gaze on imitative and spatial compatibility. The general model was as follows: DV ~ Gaze + AQ + Gaze*AQ + (1|Participant), where 'Gaze' (categorical predictor with 2 levels: direct, averted), 'AQ' (continuous predictor; mean-centred) and the interaction between the two ('Gaze*AQ') were the fixed effects, and '1|Participant' added random intercepts for participants. Imitative and spatial compatibility scores computed for RT and ER were used as dependent variables. Neither the main effects nor the predicted interaction 'Gaze*AQ' reached statistical significance in any of the four models tested (all $p > .15$; Table S1).

Table S1. Results of the linear mixed models conducted according to the preregistered analyses.

	Imitative compatibility scores								Spatial compatibility scores							
	**Model 1: Reaction times**				**Model 2: Error rates**				**Model 3: Reaction times**				**Model 4: Error rates**			
	b (95% CI)	*SE*	*t*	*p*	*Odd Ratios* (95% CI)	*SE*	*t*	*p*	*b* (95% CI)	*SE*	*t*	*p*	*Odd Ratios* (95% CI)	*SE*	*t*	*p*
Gaze	5.51 (-1.94 – 12.96)	3.80	1.45	0.15	0.001 (-0.010 – 0.012)	0.006	0.24	0.81	-4.17 (-10.70 – 2.35)	3.33	-1.25	0.21	0.006 (-0.007 – 0.019)	0.006	0.95	0.34
AQ	0.47 (-0.44 – 1.39)	0.47	1.02	0.31	0.001 (-0.001 – 0.002)	0.001	0.92	0.36	-0.24 (-1.23 – 0.74)	0.50	-0.49	0.63	-0.0004 (-0.002 – 0.001)	0.001	-0.64	0.52
Gaze*AQ	-0.97 (-2.36 – 0.41)	0.71	-1.38	0.17	0.0002 (-0.002 – 0.002)	0.001	0.18	0.86	-0.56 (-1.78 – 0.65)	0.62	-0.91	0.36	-0.001 (-0.003 – 0.001)	0.001	-0.73	0.47
**Random Effects**																
σ^2	418.72				0.001				321.50				0.002			
τ_{00}	153.91 Participant				0.001 Participant				265.81 Participant				0.001 Participant			
ICC	0.27				0.43				0.45				0.09			
N	58 Participant				58 Participant				58 Participant				58 Participant			
Observations	116				116				116				116			
Marginal R^2	0.036				0.011				0.014				0.015			
Conditional R^2	0.295				0.437				0.460				0.103			

Note: *p*-values for the fixed effects calculated using Wald-statistics approximation, uncorrected. Model equation: `Compt. scores ~ Gaze + AQ + AQ*Gaze + (1|Participant) + (1|Stimulus)`. AQ: Autism Spectrum Quotient; *b*: unstandardized coefficient; *SE*: standard error; *CI*: confidence interval; *t*: test statistic coefficient; *p*: p-value; σ^2: within-group variance; τ_{00} = between-group variance; ICC = interclass correlation (ratio of between-cluster variance to total variance); N: number

Table S2. Mean and standard deviation of reaction times (ms) and error rates for each imitative and spatial compatibility condition, as a function of gaze.

		**Reaction times**				**Error rates**			
		Direct gaze		Averted gaze		Direct gaze		Averted gaze	
		M	*SD*	*M*	*SD*	*M*	*SD*	*M*	*SD*
Imitative compatibility	Compatible	485.91	96.79	492.97	95.82	0.04	0.21	0.03	0.21
	Incompatible	500.12	105.14	502.80	104.59	0.05	0.25	0.05	0.25
Spatial compatibility	Compatible	484.32	105.50	486.82	104.34	0.03	0.20	0.04	0.21
	Incompatible	501.91	95.76	509.14	94.43	0.06	0.26	0.05	0.25

Table S3. Results of the generalized linear mixed-effects models conducted for the confirmatory analyses.

	**Model 1: Reaction times**						**Model 2: Error rates**					
	b	*95% CI*	*SE*	*ß*	*t*	*p*	*Odds Ratios*	*95% CI*	*SE*	*ß*	*t*	*p*
Imitative compt.	10.23	7.57 – 12.90	1.36	0.05	7.53	**<0.001**	1.58	1.35 – 1.85	0.08	1.12	5.59	**<0.001**
Spatial compt.	22.31	19.63 – 24.99	1.37	0.10	16.34	**<0.001**	1.70	1.45 – 2.00	0.08	1.30	6.46	**<0.001**
Gaze	-4.62	-7.29 – -1.96	1.36	-0.02	-3.41	**0.001**	0.99	0.84 – 1.16	0.08	-0.03	-0.15	0.877
AQ	0.29	-4.97 – 5.54	2.68	0.01	0.11	0.915	0.97	0.92 – 1.02	0.03	-0.75	-1.09	0.274
Gaze * Imitative	2.51	-2.75 – 7.78	2.69	0.01	0.93	0.350	0.94	0.68 – 1.29	0.16	-0.08	-0.39	0.698
Gaze * Spatial	-5.10	-10.44 – 0.23	2.72	-0.01	-1.87	0.061	1.12	0.81 – 1.54	0.16	0.14	0.67	0.503
AQ * Imitative	0.45	-0.04 – 0.94	0.25	0.01	1.79	0.074	1.03	1.00 – 1.06	0.02	0.36	1.79	0.073
AQ * Spatial	-0.15	-0.64 – 0.34	0.25	-0.004	-0.59	0.552	1.01	0.98 – 1.04	0.02	0.10	0.50	0.614
AQ * Gaze	-0.08	-0.57 – 0.41	0.25	-0.002	-0.31	0.755	1.01	0.98 – 1.04	0.02	0.14	0.69	0.490
AQ * Gaze * Imitative	-1.29	-2.28 – -0.31	0.50	-0.02	-2.59	**0.010**	1.00	0.94 – 1.06	0.03	-0.01	-0.06	0.953
AQ * Gaze * Spatial	0.16	-0.83 – 1.14	0.50	0.002	0.31	0.754	0.97	0.91 – 1.03	0.03	-0.21	-1.05	0.294
**Random Effects**												
σ^2	0.0001						3.29					
τ_{00}	32.25 Stimulus, 620.88 Participant						0.03 Stimulus, 1.00 Participant					
ICC	-						0.24					
N	60 Participant, 64 Stimulus						60 Participant, 64 Stimulus					
Observations	14047						15353					
Marginal R^2/ Conditional R^2	-						0.035 / 0.265					

Note: *p*-values for the fixed effects calculated using Wald-statistics approximation, uncorrected. Inter-class correlation (ICC) and R^2 coefficients are not reported for Model 1 as available methods for the estimation of such parameters are not optimized for GLMM with inverse-Gaussian distributions (Nakagawa, Johnson & Schielzeth, 2017). Model equation: `DV ~ Imitative + Spatial + Gaze + AQ + Gaze*Imitative + Gaze*Spatial + AQ*Imitative + AQ*Spatial + AQ*Gaze + AQ*Gaze*Imitative + AQ*Gaze*Spatial + (1|Participant) + (1|Stimulus)`. AQ: Autism Spectrum Quotient; *b*: unstandardized coefficient; *SE*: standard error; *ß*: standardized coefficient; *CI*: confidence interval; *t*: test statistic coefficient; *p*: p-value; σ^2: within-group variance; τ_{00} = between-group variance; ICC = interclass correlation (ratio of between-cluster variance to total variance); N: number of random effects.

Table S4. Results of the generalized linear mixed-effects model used to explore the influence of gaze and autistic traits on general compatibility.

	Reaction times					
	b	*95% CI*	*SE*	*ß*	*t*	*p*
General compt.	33.11	29.45 – 36.77	1.87	0.15	17.73	**<0.001**
Gaze	-4.95	-8.58 – -1.33	1.85	-0.02	-2.68	**0.007**
AQ	0.11	-5.27 – 5.49	2.75	0.01	0.04	0.967
Gaze * General compt.	-2.65	-9.87 – 4.58	3.69	-0.01	-0.72	0.473
AQ * General compt.	0.22	-0.45 – 0.89	0.34	0.01	0.64	0.524
AQ * Gaze	-0.56	-1.24 – 0.11	0.34	-0.01	-1.65	0.100
AQ * Gaze * General compt.	-1.34	-2.68 – 0.01	0.69	-0.02	-1.95	0.051
**Random Effects**						
σ^2	0.0001					
τ_{00}	50.81 $_{\text{Stimulus}}$, 857.63 $_{\text{Participant}}$					
N	60 $_{\text{Participant}}$, 64 $_{\text{Stimulus}}$					
Observations	7010					

Note: *p*-values for the fixed effects calculated using Wald-statistics approximation, uncorrected. Inter-class correlation (ICC) and R^2 coefficients are not reported as available methods for the estimation of such parameters are not optimized for GLMM with inverse-Gaussian distributions (Nakagawa, Johnson & Schielzeth, 2017). Model equation: `RT ~ General compt. + Gaze + AQ + Gaze*General compt. + AQ*General compt. + AQ*Gaze + AQ*Gaze*General compt. + (1|Participant) + (1|Stimulus)`. AQ: Autism Spectrum Quotient; *b*: unstandardized coefficient; *SE*: standard error; *ß*: standardized coefficient; *CI*: confidence interval; *t*: test statistic coefficient; *p*: p-value; σ^2: within-group variance; τ_{00} = between-group variance; ICC = interclass correlation (ratio of between-cluster variance to total variance); N: number of random effects.

Table S5. Results of the generalized linear mixed-effects model used to explore the influence of ethnicity and gaze on imitative and spatial compatibility.

	Reaction times					
	b	*95% CI*	*SE*	*ß*	*t*	*p*
Imitative compt.	10.30	7.64 – 12.95	1.36	0.05	7.60	**<0.001**
Spatial compt.	22.29	19.65 – 24.92	1.34	0.10	16.57	**<0.001**
Gaze	-4.65	-7.30 – -2.00	1.35	-0.02	-3.44	**0.001**
Ethnicity	1.02	-4.22 – 6.26	2.67	0.01	0.38	0.702
Gaze * Imitative	2.45	-2.68 – 7.59	2.62	0.01	0.94	0.350
Gaze * Spatial	-5.06	-10.20 – 0.08	2.62	-0.01	-1.93	0.054
Ethnicity * Imitative	-3.00	-8.22 – 2.22	2.66	-0.01	-1.13	0.260
Ethnicity * Spatial	-4.18	-9.42 – 1.07	2.68	-0.01	-1.56	0.118
Ethnicity * Gaze	4.01	-1.23 – 9.26	2.68	0.01	1.50	0.134
Ethnicity * Gaze * Imitative	13.72	4.20 – 23.25	4.86	0.02	2.82	**0.005**
Ethnicity * Gaze * Spatial	-3.54	-12.74 – 5.66	4.69	-0.004	-0.75	0.451
**Random Effects**						
σ^2	0.0001					
τ_{00}	32.13 $_{\text{Stimulus}}$, 621.05 $_{\text{Participant}}$					
N	60 $_{\text{Participant}}$, 64 $_{\text{Stimulus}}$					
Observations	14047					

Note: *p*-values for the fixed effects calculated using Wald-statistics approximation, uncorrected. Inter-class correlation (ICC) and R^2 coefficients are not reported as available methods for the estimation of such parameters are not optimized for GLMM with inverse-Gaussian distributions (Nakagawa, Johnson & Schielzeth, 2017). Model equation: `RT ~ Imitative + Spatial + Gaze + Ethnicity + Gaze*Imitative + Gaze*Spatial + Ethnicity*Imitative + Ethnicity*Spatial + Ethnicity*Gaze + Ethnicity*Gaze*Imitative + Ethnicity*Gaze*Spatial + (1|Participant) + (1|Stimulus)`. *b*: unstandardized coefficient; *SE*: standard error; *ß*: standardized coefficient; *CI*: confidence interval; *t*: test statistic coefficient; *p*: p-value; σ^2: within-group variance; τ_{00} = between-group variance; N: number of random effects.

Table S6. Results of the generalized linear mixed-effects models used to explore the modulation of social anxiety traits on the influence of gaze on imitative and spatial compatibility.

	**Model 1: SIAS**						**Model 2: SPS**						**Model 3: GARS**					
	b	*95% CI*	*SE*	*ß*	*t*	*p*	*b*	*95% CI*	*SE*	*ß*	*t*	*p*	*b*	*95% CI*	*SE*	*ß*	*t*	*p*
Imitative compt.	10.26	7.60 – 12.92	1.36	0.05	7.56	**<0.001**	10.16	7.49 – 12.83	1.36	0.05	7.46	**<0.001**	10.29	7.64 – 12.94	1.35	0.05	7.61	**<0.001**
Spatial compt.	22.20	19.54 – 24.86	1.36	0.10	16.36	**<0.001**	22.21	19.53 – 24.88	1.36	0.10	16.30	**<0.001**	22.28	19.64 – 24.91	1.34	0.10	16.58	**<0.001**
Gaze	-4.57	-7.23 – -1.90	1.36	-0.02	-3.36	**0.001**	-4.47	-7.15 – -1.80	1.36	-0.02	-3.28	**0.001**	-4.62	-7.27 – -1.97	1.35	-0.02	-3.42	**0.001**
Quest.	-0.69	-3.27 – 1.88	1.31	-0.08	-0.53	0.598	-1.21	-4.83 – 2.40	1.84	-0.09	-0.66	0.511	0.34	-1.76 – 2.44	1.07	0.05	0.32	0.750
Gaze * Imitative	2.97	-2.36 – 8.29	2.72	0.01	1.09	0.275	2.91	-2.33 – 8.15	2.67	0.01	1.09	0.277	2.50	-2.66 – 7.67	2.63	0.01	0.95	0.342
Gaze * Spatial	-5.34	-10.61 – -0.07	2.69	-0.01	-1.99	**0.047**	-5.15	-10.48 – 0.19	2.72	-0.01	-1.89	0.059	-5.13	-10.40 – 0.14	2.69	-0.01	-1.91	0.057
Quest.* Imitative	0.05	-0.16 – 0.26	0.11	0.003	0.47	0.639	0.18	-0.12 – 0.48	0.15	0.01	1.17	0.241	-0.04	-0.21 – 0.13	0.09	-0.003	-0.45	0.653
Quest.* Spatial	0.12	-0.08 – 0.33	0.11	0.01	1.16	0.244	0.17	-0.13 – 0.47	0.15	0.01	1.14	0.255	0.05	-0.12 – 0.22	0.09	0.003	0.56	0.576
Quest.* Gaze	-0.08	-0.29 – 0.13	0.11	-0.004	-0.78	0.435	-0.23	-0.53 – 0.07	0.15	-0.01	-1.50	0.134	-0.06	-0.23 – 0.11	0.09	-0.004	-0.72	0.471
Quest.* Gaze * Imitative	-0.68	-1.09 – -0.26	0.21	-0.02	-3.19	**0.001**	-0.72	-1.32 – -0.13	0.30	-0.01	-2.38	**0.017**	-0.36	-0.70 – -0.01	0.17	-0.01	-2.05	**0.041**
Quest.* Gaze * Spatial	0.22	-0.20 – 0.64	0.21	0.01	1.04	0.299	0.02	-0.58 – 0.62	0.31	0.0001	0.07	0.947	0.15	-0.19 – 0.49	0.17	0.01	0.88	0.379
**Random Effects**																		
σ^2	0.0001						0.0001						0.0001					
τ_{00}	31.80 Stimulus, 651.78 Participant						31.79 Stimulus, 649.58 Participant						31.63 Stimulus, 655.59 Participant					
N	60 Participant, 64 Stimulus						60 Participant, 64 Stimulus						60 Participant, 64 Stimulus					
Observations	14047						14047						14047					

Note: The predictor "Quest." refers to the corresponding questionnaire of social anxiety (SIAS, SPS or GARS). *p*-values for the fixed effects calculated using Wald-statistics approximation, uncorrected. Inter-class correlation (ICC) and R^2 coefficients are not reported as available methods for the estimation of such parameters are not optimized for GLMM with inverse-Gaussian distributions (Nakagawa, Johnson & Schielzeth, 2017). Model equation: `RT ~ Imitative + Spatial + Gaze + Questionnaire + Gaze*Imitative + Gaze*Spatial + Questionnaire*Imitative + Questionnaire*Spatial + Questionnaire*Gaze + Questionnaire*Gaze*Imitative + Questionnaire *Gaze*Spatial + (1|Participant) + (1|Stimulus).` SIAS: Social Interaction Anxiety Scale; SPS: Social Phobia Scale; GARS: Gaze Anxiety Rating Scale; *b*: unstandardized coefficient; *SE*: standard error; *ß*: standardized coefficient; *CI*: confidence interval; *t*: test statistic coefficient; *p*: p-value; σ^2: within-group variance; τ_{00} = between-group variance; N: number of random effects.

Table S7. Results of the generalized linear mixed-effects model used to explore the modulation of AQ and SIAS on the effect of gaze on imitative compatibility.

	**Reaction times**					
	b	*95% CI*	*SE*	*ß*	*t*	*p*
Imitative compt.	10.44	7.75 – 13.14	1.37	0.05	7.60	**<0.001**
Gaze	-4.61	-7.30 – -1.92	1.37	-0.02	-3.36	**0.001**
SIAS	-1.38	-4.72 – 1.97	1.71	-0.15	-0.81	0.420
AQ	2.23	-4.99 – 9.46	3.69	0.11	0.61	0.545
Gaze * Imitative	2.82	-2.47 – 8.11	2.70	0.01	1.04	0.296
SIAS * Imitative	-0.14	-0.41 – 0.13	0.14	-0.01	-1.00	0.318
SIAS * Gaze	-0.10	-0.37 – 0.17	0.14	-0.01	-0.71	0.480
AQ * Imitative	0.60	-0.04 – 1.24	0.33	0.01	1.85	0.065
AQ * Gaze	0.09	-0.55 – 0.72	0.32	0.002	0.27	0.786
SIAS * Gaze * Imitative	-0.54	-1.08 – -0.00	0.27	-0.02	-1.97	**0.048**
AQ * Gaze * Imitative	-0.56	-1.82 – 0.71	0.65	-0.01	-0.86	0.388
**Random Effects**						
σ_2	0.0001					
τ_{00}	30.82 $_{Stimulus}$, 641.04$_{Participant}$					
N	60 $_{Participant}$, 64 $_{Stimulus}$					
Observations	14047					

Note: *p*-values for the fixed effects calculated using Wald-statistics approximation, uncorrected. Inter-class correlation (ICC) and R_2 coefficients are not reported as available methods for the estimation of such parameters are not optimized for GLMM with inverse-Gaussian distributions (Nakagawa, Johnson & Schielzeth, 2017). Model equation: `RT ~ Imitative + Gaze + SIAS + AQ + Gaze*Imitative + SIAS*Imitative + SIAS*Gaze + AQ*Imitative + AQ*Gaze + SIAS*Gaze*Imitative + AQ*Gaze*Spatial + (1|Participant) + (1|Stimulus)`. *b*: unstandardized coefficient; *SE*: standard error; *ß*: standardized coefficient; *CI*: confidence interval; *t*: test statistic coefficient; *p*: p-value; σ_2: within-group variance; τ_{00} = between-group variance; N: number of random effects.

Table S8. Descriptive statistics, internal consistency reliability, and correlations between questionnaires of autistic traits and social anxiety.

Questionnaire	*M*	*SD*	Range	α	1	2	3
1. AQ	9.47	5.41	1 – 25	.82			
2. SIAS	26.43	12.15	4 – 59	.89	.64*** [.46, .77]		
3. SPS	12.20	8.67	2 – 43	.88	.44*** [.21, .64]	.68*** [.49, .81]	
4. GARS	22.72	14.87	0 – 67	.94	.29* [.04, .52]	.59*** [.39, .74]	.50*** [.30, .67]

Note. Cronbach's alpha was used as an index of internal consistency. Correlation coefficients were computed with Spearman's rank correlations. Values in square brackets indicate the 95% confidence interval for each correlation. *M*: mean; *SD*: standard deviation; α: Cronbach's alpha; AQ: Autism Spectrum Quotient (33-items version; Freitag et al., 2007); SIAS: Social Interaction Anxiety Scale (Stangier et al., 1999); SPS: Social Phobia Scale (Stangier et al., 1999); GARS: Gaze Anxiety Rating Scale (Domes et al., 2016); * $p < .05$; ** $p < .01$; *** $p < .001$.

Table S9. Summary and t-test statistics for the ratings on the attributed meaning of direct and averted gaze.

	Mean (*SD*)		T-test	Cohen's *d*
	Direct gaze	Averted gaze		
Observed	1.73 (1.29)	0.30 (0.65)	$t(59) = 8.33$, 95% *CI* [1.09, 1.78], $p < .001$	1.08
Connected	1.55 (1.23)	0.57 (0.77)	$t(59) = 7.15$, 95% *CI* [0.71, 1.26], $p < .001$	0.92
Accepted	1.30 (1.14)	0.45 (0.70)	$t(59) = 6.24$, 95% *CI* [0.58, 1.12], $p < .001$	0.81
Distracted	2.12 (1.12)	1.28 (1.03)	$t(59) = 5.05$, 95% *CI* [0.50, 1.16], $p < .001$	0.65
Pressured	1.32 (1.11)	0.67 (0.95)	$t(59) = 3.95$, 95% *CI* [0.32, 0.98], $p = .001$	0.51
Ignored	0.28 (0.67)	1.27 (1.18)	$t(59) = -6.27$, 95% *CI* [-1.30, -0.67], $p < .001$	0.81
Rejected	0.52 (0.77)	1.03 (0.94)	$t(59) = -3.60$, 95% *CI* [-0.80, -0.23], $p = .001$	0.46
Relieved	0.93 (1.09)	1.12 (1.11)	$t(59) = -1.02$, 95% *CI* [-0.54, 0.18], $p = .31$	0.13

Note. P-values were adjusted based on the Holm-Bonferroni method to correct for multiple comparisons.
